# Sexual activity and concerns in people with coronary heart disease from a population-based study

**DOI:** 10.1136/heartjnl-2015-308993

**Published:** 2016-04-28

**Authors:** Andrew Steptoe, Sarah E Jackson, Jane Wardle

**Affiliations:** Department of Epidemiology and Public Health, University College London, London, UK

## Abstract

**Objective:**

Sexual activity is a central component of intimate relationships, but sexual function may be impaired by coronary heart disease (CHD). There have been few representative population-based comparisons of sexual behaviour and concerns in people with and without CHD. We therefore investigated these issues in a large nationally representative sample of older people.

**Methods:**

We analysed cross-sectional data from 2979 men and 3711 women aged 50 and older from the English Longitudinal Study of Ageing. Sexual behaviour and concerns were assessed by validated self-completion questionnaire and analyses were weighted for non-response. Covariates included age, partnerships status and comorbidities.

**Results:**

There were 376 men and 279 women with CHD. Men with CHD were less likely to be sexually active (68.7% vs 80.0%, adjusted OR 0.62, 95% CI 0.47 to 0.81), thought less about sex (74.7% vs 81.9%, OR 0.72, CI 0.54 to 0.95), and reported more erectile difficulties (47.4% vs 38.1%, OR 1.46, CI 1.10 to 1.93) than men without CHD. Effects were more pronounced among those diagnosed within the past 4 years. Women diagnosed <4 years ago were also less likely to be sexually active (35.4% vs 55.6%, OR 0.44, CI 0.23 to 0.84). There were few differences in concerns about sexual activity. Cardiovascular medication showed weak associations with erectile dysfunction.

**Conclusions:**

There is an association between CHD and sexual activity, particularly among men, but the impact of CHD is limited. More effective advice after diagnosis might reverse the reduction in sexual activity, leading to improved quality of life.

## Introduction

There is growing interest in sexual behaviour and sexual concerns in people with coronary heart disease (CHD).^1^ ^2^ Sexual activity is a central component of intimate relationships, and impaired sex life can reduce quality of life and increase risk of depression.^3–5^ Advice given to patients about resumption of sexual activity after acute cardiac events or surgery is variable, and many patients fear that sexual activity might damage cardiac health or even cause acute events.^1^ ^6^ ^7^

Reduced sexual activity and satisfaction, problems with erections and difficulty achieving orgasm have been described in CHD.^8–12^ However, many studies have used samples recruited from a single centre or have not included appropriate age-matched comparison groups. CHD typically occurs at older ages when frequency of sexual activity is reduced compared with earlier years, and problems such as erectile dysfunction and reduced capacity for sexual arousal in women become more common.^3^ ^13^ ^14^ Studies that have included comparison groups without CHD have often shown little specific association between CHD and sexual dysfunction.^10^ ^13^

There is a need for high-quality evidence from representative population studies of older people comparing individuals with and without CHD. We therefore carried out a detailed study of sexual activity, sexual behaviour and concern about sex in the English Longitudinal Study of Ageing (ELSA), comparing participants with and without CHD, taking age, partnership status and comorbidities into account. Cardiovascular medication may be relevant to the sexual activity of people with CHD, particularly in relation to erectile function in men,^13^ ^15^ though data on women are more limited.^16^ We hypothesised that when comparisons are made with men and women without CHD of similar age, and when partnership status and comorbid health problems are taken into account, there would be few aspects of sexual activity that would be impaired in CHD. We also compared individuals with a diagnosis of CHD within the past 4 years with those who had CHD for 4 or more years, in order to test whether sexual difficulties would be more common in people with recent diagnoses.

## Methods

### Study population

ELSA is a longitudinal panel study of men and women aged 50 or more living in England that started in 2002. The sample is assessed on a two yearly basis, and these data were collected in wave 6 (2012/2013). The sample is periodically refreshed to ensure the full age range is maintained, and comparisons of sociodemographic characteristics with the national census show that the sample is representative of the English population.^17^ The general methods of data collection are detailed at http://www.elsa-project.ac.uk. The Sexual Relationships and Activities Questionnaire (SRA-Q) was administered as a self-completion measure and was returned by 7079 (67%) participants. We excluded individuals who failed to state whether or not they had been sexually active over the last year, and respondents who did not have CHD but had a history of stroke or heart failure, since these conditions may have a shared aetiology with CHD. The study sample therefore consisted of 6690 respondents, 2979 men and 3711 women. The study was approved by the National Research Ethics Service, and all participants provided informed signed consent.

### Definition of CHD

The presence of CHD was defined as a doctor diagnosis of myocardial infarction (MI) or angina pectoris. Participants provided this information at biennial waves of data collection, so the CHD cases were subsequently divided into those who had been diagnosed within the past 4 years or ≥4 years ago.

### Sexual activity, sexual behaviour and sexual concerns

The SRA-Q has been described in detail elsewhere.^3^ The questionnaire was derived from previously validated measures with modifications to ensure comparability with the National Social Life, Health and Aging Study in the USA^18^ and with the National Survey of Sexual Attitudes and Lifestyles in the UK.^12^ It covers a wide range of information on attitudes to sex; frequency of sexual activities and behaviours; problems with sexual activities and function; concerns and worries about sexual activities and sexual function; and satisfaction with sex. The male and female versions of the SRA-Q are available online at http://www.elsa-project.ac.uk/documentation. Participants completed the questionnaire in private and returned it in a sealed envelope. Details of the items presented in this report are provided in the online supplementary material.

10.1136/heartjnl-2015-308993.supp1 Supplementary data

### Other variables

Partnership status was defined as whether or not the respondent had a spouse or partner at the time of wave 6 data collection. We included physician diagnosed diabetes as a covariate, together with a comorbidity index that summed physician diagnoses of cancer of any type, arthritis of any type and chronic lung disease within the last 4 years. Objective information about medication was obtained during a visit by a research nurse to the participants’ homes in which details of prescribed medicines were recorded.

### Statistical analysis

We used weights to correct for sampling probabilities and for differential non-response and to calibrate back to the 2011 National Census population distributions for age and sex. The weights accounted for the differential probability of being included in Wave 6 of ELSA, and for non-response to the SRA-Q. Details can be found in http://doc.ukdataservice.ac.uk/doc/5050/mrdoc/pdf/5050_elsa_w6_technical_report_v1.pdf. We used logistic regression to analyse the association between CHD and sexual activities and concerns, with age, partnership status, diabetes and number of comorbidities as covariates. Both age and comorbidities were modelled as continuous variables. Separate analyses were carried out on men and women. The results are presented as age-adjusted percentages and adjusted ORs, with 95% CIs. The no CHD group was the reference category. Within the CHD group, we tested selected associations with medication as detailed in the Results section. All analyses were repeated on MI cases alone; results were similar, so are only described when they differed from the complete CHD sample. In addition, we carried out two sensitivity analyses. First, we stratified the analysis by marital/partnership status. Second, we excluded participants with diabetes.

## Results

There were 2979 men and 3711 women in the study, of whom 376 (12.6%) men and 279 (7.5%) women had CHD. CHD had been diagnosed 4 or more years ago in 294 (78.2%) men and 211 (75.6%) women, and of these, 225 (68.4%) had been diagnosed ≥4 years previously. There were 329 MI diagnoses among the CHD cases, including 218 men and 111 women. Participants with CHD were significantly older than those without CHD (men: means 71.7±9.4 vs 65.6±8.7 years; women: means 73.8±10.0 vs 65.1±9.1 years, both p<0.001). We found that 70.7% of men and 47.7% of women with CHD were married or living with a partner, compared with 77.7% and 65.0% of men and women without CHD (p<0.001). Diabetes was more common in men and women with CHD than without CHD (23.5% vs 10.5% and 27.6% vs 8.5%, respectively, p<0.001), and the number of comorbidities was significantly higher in CHD cases in both men and women (both p<0.001). A total of 93.6% of men and 94.7% of women reported being exclusively heterosexual over their lifetimes.

### Sexual activity and CHD

The majority of men (78.6%) and women (55.1%) in this study were sexually active. But fewer men with CHD than without CHD reported any sexual activity over the past year (68.7% vs 80%, table 1). After adjustment for covariates, CHD was independently associated with a 38% reduction in the odds of reporting any sexual activity. Women with CHD were also less likely to report any sexual activity than those without CHD, but the difference was not significant. Other independent predictors of not being sexually active were older age (OR=0.89 and 0.91 for men and women), not being married (OR=0.56 and 0.21), diabetes (OR=0.53 and 0.54) and comorbidities (OR=0.76 and 0.85, all p<0.001). CHD was associated with lower rates of thinking about sex in men, a difference that was significant after adjustment for covariates (table 1, p=0.022).

**Table 1:** Sexual activity and coronary heart disease (CHD)

	Category	% adjusted for age	OR, fully adjusted* (95% CI)	p Value
Any sexual activity in the past year				
Men (n=2979)	No CHD	80.0	1	
	CHD	68.7	0.62 (0.47 to 0.81)	<0.001
Women (n=3711)	No CHD	55.6	1	
	CHD	49.2	0.91 (0.65 to 1.27)	0.58
Thinking about sex at least 2–3 times over the past month				
Men (n=2967)	No CHD	81.9	1	
	CHD	74.7	0.72 (0.54 to 0.95)	0.022
Women (n=3692)	No CHD	47.2	1	
	CHD	43.4	0.82 (0.58 to 1.15)	0.24
Sexual intercourse at least 2–3 times over the past month†				
Men (n=2275)	No CHD	48.9	1	
	CHD	43.7	0.83 (0.59 to 1.16)	0.27
Women (n=2087)	No CHD	50.0	1	
	CHD	45.7	0.82 (0.48 to 1.39)	0.46
Other sexual behaviours at least 2–3 times over the past month†				
Men (n=2277)	No CHD	64.7	1	
	CHD	58.7	0.76 (0.56 to 1.05)	0.097
Women (n=2084)	No CHD	67.9	1	
	CHD	69.0	1.05 (0.62 to 1.76)	0.87
Erectile difficulties				
Men (n=3041)	No CHD	38.1	1	
	CHD	47.4	1.46 (1.10 to 1.93)	0.009
Difficulty becoming sexually aroused‡				
Women (n=1511)	No CHD	32.1	1	
	CHD	26.0	0.91 (0.48 to 1.72)	0.77
Difficulty achieving orgasm‡				
Men (n=1625)	No CHD	12.6	1	
	CHD	21.0	1.49 (0.91 to 2.44)	0.12
Women (n=1443)	No CHD	28.2	1	
	CHD	33.8	1.38 (0.70 to 2.69)	0.35

All analyses weighted for sampling probabilities and differential non-response.

*Adjustment for age, partnership status, diabetes and number of comorbidities.

†Among participants reporting sexual activity in the past year.

‡Among participants reporting sexual activity in the past month.

Among sexually active participants, we found no differences in the frequency either of sexual intercourse or other sexual behaviours (kissing, petting or fondling) related to CHD. Women with CHD did not experience increased difficulty in becoming sexually aroused (table 1). However, CHD was associated with a 46% increase in the odds of men being never or only sometimes able to get or keep an erection good enough for sexual activity (p=0.009), adjusting for covariates. Erectile difficulties were also associated with older age (OR=1.13), diabetes (OR=2.24) and comorbidities (OR=1.33). There were no significant differences in difficulty achieving orgasm in participants with and without CHD.

### Sexual activity and years since diagnosis

The reduction in prevalence of sexual activity among participants with CHD was related to the time since diagnosis. Men who had been diagnosed 4 or more years ago had a 19% reduction in odds of being sexually active, while those diagnosed <4 years ago had a 76% decrease in odds compared with men free of CHD (table 2). Among women, there was no difference from controls in those diagnosed ≥4 years ago, but there was a 56% reduction in those with more recent diagnoses (p=0.013). Years since diagnosis was also associated with rates of thinking about sex among men, with bigger reductions in men diagnosed <4 years ago. A similar pattern was observed for men in relation to erectile difficulties (table 2), with a twofold increase in those diagnosed <4 years ago.

**Table 2:** Sexual activity and years since diagnosis of coronary heart disease (CHD)

	Category	% adjusted for age	OR, fully adjusted* (95% CI)	p Value
Any sexual activity in the past year				
Men (n=2979)	No CHD	80.0	1	
	CHD≥4 years	73.5	0.81 (0.59 to 1.10)	0.099
	CHD<4 years	53.6	0.24 (0.14 to 0.41)	<0.001
Women (n=3711)	No CHD	55.6	1	
	CHD≥4 years	54.0	1.18 (0.81 to 1.73)	0.38
	CHD<4 years	35.4	0.44 (0.23 to 0.84)	0.013
Thinking about sex at least 2–3 times over the past month				
Men (n=2967)	No CHD	81.9	1	
	CHD≥4 years	75.7	0.77 (0.57 to 1.04)	0.095
	CHD<4 years	71.8	0.55 (0.31 to 0.97)	0.039
Women (n=3692)	No CHD	47.2	1	
	CHD≥4 years	44.9	0.87 (0.59 to 1.30)	0.50
	CHD<4 years	39.0	0.69 (0.37 to 1.29)	0.25
Erectile difficulties				
Men (n=3041)	No CHD	38.1	1	
	CHD≥4 years	45.3	1.28 (0.93 to 1.77)	0.13
	CHD<4 years	54.0	2.11 (1.24 to 3.59)	0.006

All analyses weighted for sampling probabilities and differential non-response.

*Adjustment for age, partnership status, diabetes and number of comorbidities.

### Sexual concerns and CHD

Table 3 summarises findings relating concerns about sexual activity and function with CHD. There was no association between CHD and concerns about level of sexual desire, frequency of sexual activity or satisfaction with sex life. The only significant difference was that more men with CHD expressed concern about orgasmic experience. Levels of sexual concerns did not differ systematically between the groups with recent or more distant diagnosis.

**Table 3:** Coronary heart disease (CHD) and concerns about sexual activity

	Category	% adjusted for age	OR, fully adjusted* (95% CI)	p Value
Concern about level of sexual desire				
Men (n=2978)	No CHD	13.3	1	
	CHD	15.5	1.19 (0.85 to 1.67)	0.31
Women (n=3705	No CHD	7.4	1	
	CHD	5.4	0.42 (0.16 to 1.15)	0.09
Concern about frequency of sexual activity†				
Men (n=2287)	No CHD	13.1	1	
	CHD	14.7	1.07 (0.68 to 1.68)	0.77
Women (n=2097)	No CHD	7.7	1	
	CHD	7.4	0.15 (0.36 to 3.63)	0.82
Concern about orgasmic experience†				
Men (n=1963)	No CHD	10.4	1	
	CHD	17.5	1.69 (1.06 to 2.70)	0.027
Women (n=1587)	No CHD	6.7	1	
	CHD	6.5	1.39 (0.42 to 4.59)	0.59
Concern about ability to have an erection				
Men (n=2965)	No CHD	12.9	1	
	CHD	16.5	1.27 (0.93 to 1.74)	0.14
Concern about ability to become sexually aroused†				
Women (n=1655)	No CHD	7.6	1	
	CHD	3.0	0.47 (0.11 to 1.94)	0.29
Worry or concern about sex life overall				
Men (n=2934)	No CHD	18.6	1	
	CHD	24.0	1.32 (0.92 to 1.91)	0.14
Women (n=3613)	No CHD	13.3	1	
	CHD	9.7	0.83 (0.33 to 2.08)	0.69
Dissatisfaction with sex life overall‡				
Men (n=1519)	No CHD	19.9	1	
	CHD	18.9	0.90 (0.52 to 1.55)	0.69
Women (n=1534)	No CHD	12.4	1	
	CHD	8.9	0.44 (0.09 to 2.06)	0.30

*Adjustment for age, partnership status, diabetes and number of comorbidities.

†In participants reporting sexual activity in the past year.

‡Over the past 3 months.

### Sexual activity and MI

The pattern of results was largely the same when the analysis was limited to MI. The only major difference was related to erectile difficulties. Men who had experienced an MI within the past 4 years showed a greater prevalence of erectile difficulties compared with those without disease (62.1% vs 38.7%) than in the overall CHD analysis. The adjusted odds were 3.63 (CI 1.93 to 6.84, p<0.001). By contrast, men with MI ≥4 years earlier did not report excess erectile difficulty (prevalence 39.4%).

### Association with medication

The most common cardiovascular medications in the CHD group were statins (78.7%), aspirin (64.2%), ACE inhibitors (56.8%), β-blockers (49.2%), glyceryl trinitrate (31.9%) and calcium channel blockers (29.6%). We tested associations between different classes of medication and the three aspects of sexual activity that were problematic in CHD: overall sexual activity in men and women, and erectile problems and difficulty achieving orgasm in men. Two associations were significant: men prescribed diuretics were more likely to report erectile difficulties (adjusted OR 3.70, CI 1.33 to 10.33, p=0.012) as were men prescribed statins (adjusted OR 2.03, CI 1.01 to 4.09, p=0.048). There were no other associations between any class of medication and sexual activity.

Phosphodiesterase type 5 inhibitors such as sildenafil and tadalafil were rarely reported, being used by only 5.7% of men with CHD and 7.4% of those without CHD. These numbers were too small for statistical analysis.

### Sensitivity analyses

Stratification of the sample by marital/partnership status resulted in similar findings to those observed in the complete sample, with no major differences in association with CHD in the two groups. Excluding people with diabetes did not markedly alter the results with respect to CHD.

## Discussion

This study of sexual behaviour and concerns about sexual function showed that men with CHD were less likely to have been sexually active over the past year, thought less about sex and reported more erectile problems than men without CHD. Differences remained significant after adjusting for age, partner status and comorbidities, and were more pronounced among those diagnosed in the past 4 years than in men with longer standing disease. Women diagnosed within the past 4 years were less likely to have been sexually active than those diagnosed ≥4 years ago. There were few differences in sexual satisfaction or concerns about sexual activity and function, and cardiovascular medication showed limited associations with sexual function. Results were similar when the analyses were restricted to people who had experienced an MI, suggesting that associations are more dependent on the presence of CHD than on its clinical presentation. Both marital status and diabetes were related to sexual behaviour, but did not affect the associations with CHD.

The study involved a large representative sample of men and women aged 50 and over living in England, with 8.7% men and 8.0% women being aged 80 or older. The prevalence of CHD was 12.6% in men and 7.5% in women, similar to those observed nationally in the UK.^19^ As expected, CHD cases were older on average than the remainder of the sample, and were less likely to be married or in a partnership, and had greater comorbidities. These factors are related to sexual behaviour,^3^ ^13^ ^14^ so these were taken into account in the analyses. The response rate of 67% to the SRA-Q was high, considering the intimate nature of the items, and we weighted our analyses to take account of differential non-response.

The most striking difference was the reduced proportion of respondents with CHD who were sexually active. This was particularly evident among those with recently diagnosed CHD. We found age-adjusted differences of 26.4% in men and 20.2% in women between no CHD and respondents who had been diagnosed <4 years. However, sexual activity was still common in the latter group, with over half men and a third of women being sexually active.

Among those who were sexually active, we found no differences in the frequency of intercourse and other sexual behaviour. Additionally, the differences in concern about different aspects of sex between people with and without CHD were small. This strongly suggests that the primary issue among older people with CHD is whether or not they resume sexual activity following diagnosis, not the behaviours of those who are sexually active. Quantitative and qualitative research indicates that only a minority of patients receive advice about resumption of sexual activity following MI or cardiac surgery,^6^ ^7^ ^20^ despite guidance issued by the European Society of Cardiology and the American Heart Association.^1^ ^2^ A large multicentre study of younger patients showed that many patients are given advice about resuming sexual activity after acute MI that is not consistent with guidelines, including recommendations to limit the frequency of sex and take a passive role.^7^ There is a significant but very small risk that sexual activity can trigger acute cardiac events,^21^ and beliefs about such effects may be magnified in patients and their families unless healthcare staff provide appropriate advice.

Rates of sexual activity in ELSA are comparable with other contemporary studies of older people in the UK and Europe.^12^ ^22^ Sexual activity rates in people with CHD have varied substantially across studies.^9^ Addis *et al*^23^ assessed a large sample of women aged 50 and older with CHD in 1994–1996 and found that only 39% were sexually active, compared with 49% in the present study. An analysis from the Women's Health Initiative showed that 52% of those aged 50–79 were sexually active, with no differences related to CHD.^10^ Other researchers have reported greater dissatisfaction with sex among people with CHD than those measured in the present study,^8^ ^10^ but differences in measures and in the era in which data were collected make direct comparisons difficult.

We investigated the possible role of cardiac medication, but the only significant relationships were between erectile dysfunction and prescription of diuretics and statins. The association with diuretics has been observed in previous studies,^15^ ^24^ but the statin effect has not. Since we tested several aspects of sexual behaviour and many different medications, the two significant associations may be chance effects. We were not able to analyse associations between phosphodiesterase type 5 inhibitor use and sexual activity because of small numbers.

This study has a number of strengths. We collected data within a large well-characterised nationally representative sample of older men and women. We assessed sexual behaviour and concerns with a detailed multidimensional inventory, in contrast with studies that have asked one or two broad questions.^8^ ^10^ ^23^ We ascertained medication by direct examination of prescription medication rather than relying on self-report. Because the data were embedded in a longitudinal study, we were able to ascertain the length of time since diagnosis. However, the study was cross-sectional so no causal conclusions can be drawn, and our data are not able to contribute to the debate concerning erectile dysfunction and cardiovascular disease risk.^25^ The time frame for different aspects of sexual behaviour and concerns was dictated by the structure of the SRA-Q. The division into recent (<4 years) and more distant CHD diagnoses was determined by the data collection schedule of ELSA and may not have been optimal. As with most large-scale observational studies, CHD was based on reported physician diagnoses. This may result in some error, although the evidence from direct comparisons is that little bias is introduced.^26^ ^27^

Sex is an important feature of many intimate relationships, and sexual difficulties can be a major source of interpersonal conflict and marital stress, contributing to reduced quality of life.^5^ These factors are important in CHD, since marital tension and reduced social support may augment risk of recurrent cardiac events.^28^ ^29^ Our findings are generally encouraging in showing little increased risk of concerns about sex in either men or women or sexual difficulties in women with CHD. However, the results do indicate that CHD is associated with a reduced proportion of people who are sexually active, particularly among those with a diagnosis in the past 4 years. More effective advice about sexual activity after diagnosis might reverse this pattern, leading to more satisfying personal lives. The heightened risk of erectile dysfunction and associated concerns in men with CHD requires focused advice and active management.^1^

**Key messages**

**What is already known on this subject?**

- Sexual activity can be adversely affected by coronary heart disease (CHD). But few studies have investigated sexual behaviour in nationally representative samples involving similarly aged comparison groups.

**What might this study add?**

- Men aged 50 and over with CHD were less likely to be sexually active and reported more erectile problems than those without CHD, with associations being more pronounced among those diagnosed in the past 4 years. There were few differences in women, and sexual satisfaction and concerns about sex appeared unaffected in both men and women.

**How might this impact on clinical practice?**

- Systematic advice about resumption of sexual activity after diagnosis might restore sexual activity more rapidly, potentially benefiting close relationships.
