# Supplementary material for: Sexual activity and concerns in people with coronary heart disease from a population-based study
Source: Heart. 2016 Apr 28;102(14):1095–9. doi: 10.1136/heartjnl-2015-308993 (PMC4941181; doi:10.1136/heartjnl-2015-308993)
Supplement: Supplementary data [file heartjnl-2015-308993supp.pdf]

Supplemental table: Measures of sexual behaviour, sexual function, and sexual concerns

Item*	Response options	Classification
**Sexual behaviour and activities**		
Have you had any sexual activity (sexual intercourse, masturbation, petting or fondling) in the past year?	Yes / No	Yes / no
How often did you think about sex?	7-point scale: ‘not at all’ to ‘more than once a day’	Thinking about sex frequently: ‘2–3 times a month’ or more
For participants who reported sexual activity in the past year:		
How many times have you had or attempted sexual intercourse (vaginal, anal or oral)?	7-point scale: ‘not at all’ to ‘more than once a day’	Frequent sexual intercourse: ‘2–3 times a month’ or more
How frequently did you engage in other sexual activities (kissing, petting or fondling)?	7-point scale: ‘not at all’ to ‘more than once a day’	Frequent kissing, petting, or fondling: ‘2–3 times a month’ or more
**Sexual function**		
Are you able to get or keep an erection which would be good enough for sexual activity? [men]	4-point scale: ‘always able’ to ‘never able’	Erectile difficulties: ‘never able’ or ‘sometimes able’
For participants who reported sexual activity in the past month:		
How often did you feel sexually aroused during sexual activity? [women]	5-point scale: ‘never’ to ‘always’	Difficulty becoming sexually aroused: ‘never’ or ‘much less than half the time’
When you had sexual stimulation how difficult was it for you to reach orgasm?	5-point scale: ‘impossible’ to ‘not at all’	Difficulty achieving orgasm: ‘moderately difficult’ to ‘impossible’
**Sexual concerns**		
Have you been worried or concerned by your level of sexual desire?	5-point scale: ‘not at all worried or concerned’ to ‘extremely worried or concerned’	Concerned about level of sexual desire: ‘moderately’, ‘very’, or ‘extremely worried or concerned’
Have you been worried or concerned by your ability to have an erection? [men]	5-point scale: ‘not at all worried or concerned’ to ‘extremely worried or concerned’	Concerned about ability to have an erection: ‘moderately’, ‘very’, or ‘extremely worried or concerned’
Even if you have not had any sexual activity, during the past three months, how worried or concerned have you	5-point scale: ‘not at all worried or concerned’ to ‘extremely worried or concerned’	Concerned about overall sex life: ‘moderately’, ‘very’, or ‘extremely worried or concerned’

Item*	Response options	Classification
been about your overall sex life?		
For participants who reported sexual activity in the past year:		
Have you been worried or concerned by the frequency of your sexual activities?	5-point scale: ‘not at all worried or concerned’ to ‘extremely worried or concerned’	Concerned about frequency of sexual activities: ‘moderately’, ‘very’, or ‘extremely worried or concerned’
Are you worried or concerned by your current ability to become sexually aroused? [women]	5-point scale: ‘not at all worried or concerned’ to ‘extremely worried or concerned’	Concerned about ability to become sexually aroused: ‘moderately’, ‘very’, or ‘extremely worried or concerned’
Have you been worried or concerned by your orgasmic experience?	5-point scale: ‘not at all worried or concerned’ to ‘extremely worried or concerned’	Concerned about orgasmic experience: ‘moderately’, ‘very’, or ‘extremely worried or concerned’
**Sexual satisfaction (during the past three months)**		
How satisfied have you been with your overall sex life?	5-point scale: ‘very satisfied’ to ‘very dissatisfied’	Dissatisfied with overall sex life: ‘moderately dissatisfied’ or ‘very dissatisfied’

*All items were asked of both men and women unless otherwise specified.
